# PhosphoregDB: The tissue and sub-cellular distribution of mammalian protein kinases and phosphatases

**DOI:** 10.1186/1471-2105-7-82

**Published:** 2006-02-20

**Authors:** Alistair RR Forrest, Darrin F Taylor, J Lynn Fink, M Milena Gongora, Cameron Flegg, Rohan D Teasdale, Harukazu Suzuki, Mutsumi Kanamori, Chikatoshi Kai, Yoshihide Hayashizaki, Sean M Grimmond

**Affiliations:** 1 The Institute for Molecular Bioscience, University of Queensland, Brisbane 4072, Australia; 2 The Australian Research Council Centre in Bioinformatics, University of Queensland, Brisbane 4072, Australia; 3 Genome Exploration Research Group (Genome Network Project Core Group), RIKEN Genomic Sciences Center (GSC), RIKEN Yokohama Institute, Yokohama, Kanagawa, 230-0045, Japan; 4 Genome Science Laboratory, Discovery Research Institute, RIKEN Wako Institute, Wako, Saitama, 351-0198, Japan

## Abstract

**Background:**

Protein kinases and protein phosphatases are the fundamental components of phosphorylation dependent protein regulatory systems. We have created a database for the protein kinase-like and phosphatase-like loci of mouse  that integrates protein sequence, interaction, classification and pathway information with the results of a systematic screen of their sub-cellular localization and tissue specific expression data mined from the GNF tissue atlas of mouse.

**Results:**

The database lets users query where a specific kinase or phosphatase is expressed at both the tissue and sub-cellular levels. Similarly the interface allows the user to query by tissue, pathway or sub-cellular localization, to reveal which components are co-expressed or co-localized. A review of their expression reveals 30% of these components are detected in all tissues tested while 70% show some level of tissue restriction. Hierarchical clustering of the expression data reveals that expression of these genes can be used to separate the samples into tissues of related lineage, including 3 larger clusters of nervous tissue, developing embryo and cells of the immune system. By overlaying the expression, sub-cellular localization and classification data we examine correlations between class, specificity and tissue restriction and show that tyrosine kinases are more generally expressed in fewer tissues than serine/threonine kinases.

**Conclusion:**

Together these data demonstrate that cell type specific systems exist to regulate protein phosphorylation and that for accurate modelling and for determination of enzyme substrate relationships the co-location of components needs to be considered.

## Background

It has been estimated that more than a third of all Eukaryotic proteins are subjected to phosphorylation [1]. The phosphorylation state of a protein can regulate functional properties including bioactivity, stability, sub-cellular localization, conformation and the ability to interact with other binding partners [2-6]. In mammals, several hundred kinases and phosphatases compete in a highly dynamic and spatially restricted fashion to control these post-translational modifications [7,8].

For budding yeast 43 phosphatase and over 120 protein kinase like sequences [9] regulate processes such as cell cycle, DNA damage response, and signal transduction. For these organisms the network components of phospho-regulation are well defined and physically contained within one cell type however in multi-cellular organisms, the role of inter-cell communication and tissue specific expression comes into play. Developmental stage and lineage specific expression of these proteins is used to regulate a diverse range of multicellular processes including immune response, differentiation, and memory [10-12].

One major undertaking is the elucidation of substrate-enzyme relationships for these proteins [13]. Protein arrays, yeast-2 hybrid and phage libraries have all been used as high-throughput methods to identify likely substrates for these proteins however they are far removed from a natural cellular environment and an interaction identified here may not occur in vivo. Lower throughput screens by substrate trapping mutants and immuno-precipitation are more biologically relevant but are not realistic for mass screening of the proteome. By providing sub-cellular localization and tissue information we hope to make it easier for researchers to focus on the kinase or phosphatase most likely to be expressed in the same tissue and localized to the same compartment as the substrate of interest.

Understanding the spatial expression of the phosphoregulators is also essential to build meaningful models of a mammalian protein phosphorylation network. Many components display restricted expression, and require compartmentalization or transient association with sub-cellular structures in order to function appropriately. To address this we provide a database of the sub-cellular localization and tissue specificity of every protein kinase and phosphatase of mouse. Sub-cellular localization is provided by a combination of literature review, bioinformatic prediction and a novel high-throughput sub-cellular localization screen. Evidence for all localizations is provided and for the experimental localizations original images are displayed. For tissue specificity we provide an expression summary for the phosphoregulators in 61 normal mouse tissues by mining the expression data of the GNF gene atlas [14,15]. All of this is then combined into a simple interface that allows the user to query by gene name, tissue, compartment, pathway and classification.

## Results and Discussion

### Sub-cellular localization of mouse protein kinases and phosphatases

The sub-cellular localizations recorded in PhosphoregDB are compiled from three sources; newly reported experimental localizations from a simple PCR based tagging strategy, previously published localizations from a systematic review of the literature and bioinformatic predictions. Using these sources a consensus description of the localization was made and supporting evidence provided for each decision.

**Figure 1:**
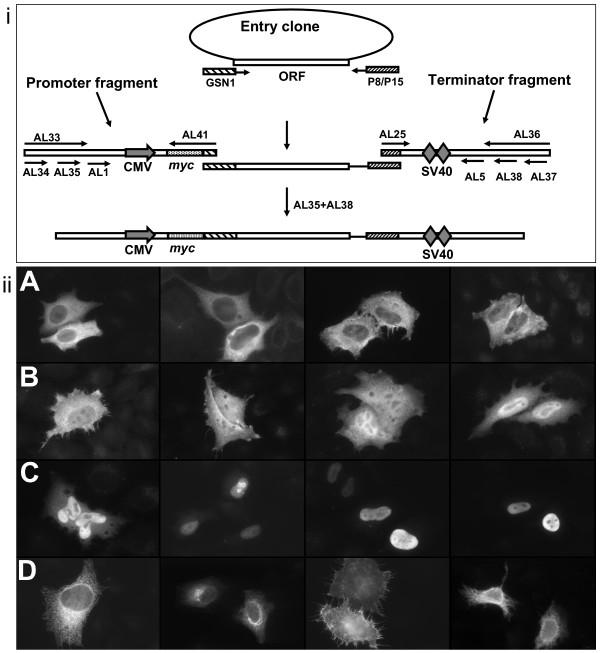
**Experimental localization**. **i**) Rapid PCR generation of tagged expression constructs. A linear expression construct is produced by fusing three fragments: a CMV promoter, a dual SV40 terminator and the *myc *tagged open reading frame to be tested. The epitope tag is added by use of a gene specific primer that fuses the epitope in-frame with the open reading frame. Overlap sequences corresponding to AL41/GSN1 and P15/AL25 are used to prime off each other to generate a full length construct consisting of all three products. Nested primers AL35 and AL38 are used to further amplify the construct. Primer sequences and C-terminal schematics available as Supplemental data. **ii**) Representative observed localizations. **A**) Cytoplasmic: (left to right) Rps6kb1, 4932415A06Rik, Camk2d, Lats2 **B**) Ubiquitous, nuclear and cytoplasmic: Csnk1a1, Camkk2, Stk35, Mapkapk3 **C**) Nuclear: Dusp4, Mastl, Smok1, Trib1 **D**) Membrane associated: Ptpn5, Txk, Ptp4a3, Vrk2. Bar 10 μm.

A systematic review of the literature and mining of entries in the public localization database DBSubLoc [16] found published localizations for 396 protein kinases and 102 protein phosphatases. We extended the number of experimentally observed localizations to 438 and 123 respectively by epitope tagging full-length open reading frames from the FANTOM2 mouse gene encyclopedia [17,18]. Using an overlap fusion PCR protocol we generated linear mammalian expression constructs from three fragments; a CMV promoter, a *myc *9E11 epitope tagged open reading frame and two copies of the SV40 mRNA polyadenylation signal. These constructs were then transfected into HeLa cells and localizations recorded by immunofluorescence after 16 hours (Fig. 1). Using this strategy we experimentally recorded the sub-cellular localizations of 109 kinase-like and 50 phosphatase-like open reading frames.

For the remaining 91 kinases and 29 phosphatases where we lacked full length clones, we used sub-cellular localization predictions from SublocV1.0, Proteome Analyst, and membrane organisation predictions from TMHMM and signalP to provide a consensus prediction of the localization [19-26].

**Figure 2:**
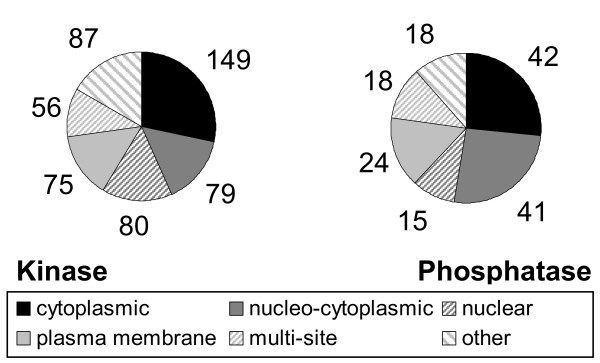
Sub-cellular distributions of mouse protein kinases and phosphatases.

By combining sub-cellular localization information from the above three approaches we produced consensus localizations for each protein. Overall the protein kinases and phosphatases displayed very similar distributions (Fig. 2). Peptides localizing to the cytoplasm, nucleus or both accounted for more than 58% of all entries. The next largest groups were peptides localizing to the plasma membrane and those with multi-site localizations. The remainder corresponds to the small numbers of peptides that localize to other structures such as the endoplasmic reticulum, golgi, and cytoskeleton.

The observation of peptides in multiple localizations, was of interest in particular the large number with nucleo-cytoplasmic distributions. Literature on these peptides reveals that of the 83 previously recorded peptides of this class, 39 actively shuttle between the nucleus and the cytoplasm and 14 of these are known to leave the nucleus in a leptomycinB sensistive/Crm1 dependent manner [27]. We record this in the annotations and additionally provide predictions of nuclear localization signals (NLS) and nuclear export sequences (NES) when found [28-33].

### Tissue specificity of mouse protein kinases and phosphatases

To provide users with an estimate of tissue specificity of each protein kinase and phosphatase we mined the Genomics Institute of the Novartis Research Foundation gene atlas [14,15] for expression of these genes across a panel of 61 normal mouse tissues. From the gene atlas we extracted GC-RMA normalised expression data for 1062 probe sequences representing 643 loci. Using a threshold for detection of 200 relative expression units as described by Su et al. 2004, 567 of the 643 loci had detectable expression in at least one tissue. The 76 loci with uninformative probes were excluded from further analysis however some of these are likely to represent tissue specific transcripts from tissues not sampled in the GNF gene atlas.

**Figure 3:**
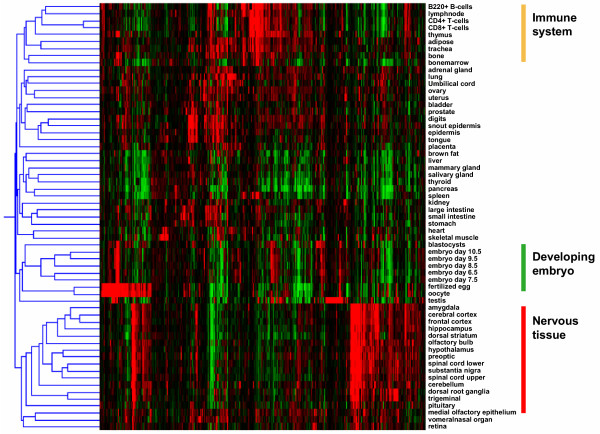
**Protein kinases and phosphatases display tissue specificity**. Hierarchical clustering of the expression patterns of 571 kinases and phosphatases across the 61 tissues of the GNF gene atlas. Genes and tissues were clustered in Genespring 6.2 using Pearson correlation.

Comparing the expression patterns of the 567 loci with signal above threshold we observed clusters of tissue specific genes. We hierarchically clustered the genes and tissues using Pearson correlation and visualised the trees using GeneSpring 6.2. This largely split the data into clusters of tissues of related lineage. Three major clusters identified were immuno-related (B220+ B-cells, lymphnode, CD4+T-cells, CD8+T-cells, thymus, bone, bonemarrow and spleen), developing embryo (stages 6.5, 7.5, 8.5, 9.5 and 10.5) and nervous tissue (amygdala, cerebral cortex, frontal cortex, hippocampus, dorsal striatum, olfactory bulb, hypothalamus, preoptic, spinal cord lower, substantia nigra, spinal cord upper, cerebellum, dorsal root ganglia and trigeminal) (Fig. 3).

**Figure 4:**
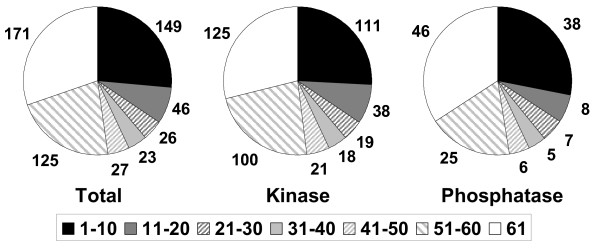
**Tissue restriction of protein kinases and phosphatases**. Counts were made of the number of tissues a given gene was detected in (above 200). Then genes were divided into bins of size 10, where bin 1–10 corresponded to genes detected in 10 or fewer tissues. Bin 61, corresponds to the fraction detected in all 61 tissues.

The overall tissue specificity of the dataset was then assessed by examining how many tissues each gene was expressed in (above 200). In total 171 of the set were detected in all 61 tissues while 40 were detected in only one tissue (15 of which were testis specific). To further assess tissue restriction we split the genes into bins based upon detection in 1–10, 11–20, 21–30, 31–40, 41–50, 51–60 or 61 tissues (Fig. 4). Using this we show that the majority of phosphoregulators show some form of restricted expression, similarly when we sub divide the set into kinase and phosphatase they show similar levels of restriction.

### Relationships between localization and classification with tissue restriction

**Figure 5:**
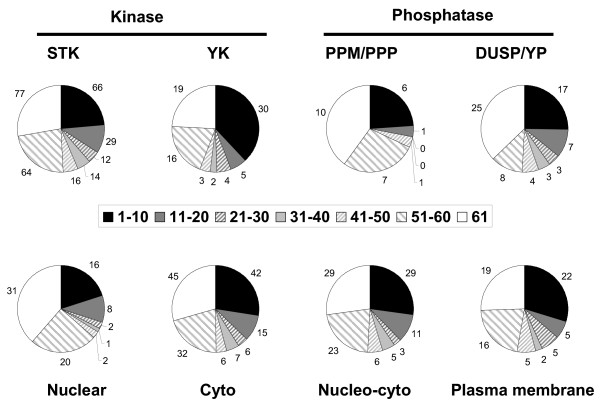
**Correlation of class and localization with tissue specificity**. Using the same bins as described in figure 4 we demonstrate the relationship between classification and sub-cellular localization with tissue specificity. **A**) Serine/threonine kinases (STK n = 278), Tyrosine kinases (YK n = 79), serine/threonine phosphatases (PPM/PPP n = 25) and dual specificity/tyrosine phosphatases (DUSP/YP n = 67) **B**) Nuclear (n = 80), Cytoplasmic (n = 153), Nucleo-cytoplasmic (n = 106), Plasma membrane (n = 74).

One question we sought to answer was whether kinases or phosphatases localizing to a particular structure or belonging to a particular class were more likely to be expressed in a restricted fashion. For example, we expected cell surface receptors used for cell to cell communication to be more likely to be expressed in a restricted pattern than those localized in the cytoplasm or nucleus. To address this question we again binned genes based upon the number of tissues they were detected in and then overlaid them with broad classifications of substrate specificity; serine/threonine kinases, tyrosine kinases, serine/threonine phosphatases of the PPP and PPM groups and dual specificity/tyrosine phosphatases (Fig. 5A). Similarly we compared number of tissues detected in with the sub-cellular localizations nuclear, cytoplasmic, plasma-membrane and nucleo-cytoplasmic (Fig. 5B).

Although the overall tissue specificity of the kinases and phosphatases is similar (Fig. 4) we identified some general trends in the tissue distribution of different classes and localizations (Fig. 5A). The greatest difference was observed between kinase classes, 38% of tyrosine as compared to 24% of serine/threonine kinases were restricted to 10 or fewer tissues (p < = 0.02). Similarly for the phosphatases, more than two thirds (68%) of serine/threonine phosphatases were widely expressed across 51 or more tissues as compared to 50% for the dual specificity and tyrosine phosphatases (p < = 0.17).

Considering proteins with different localizations (Fig. 5B), the cytoplasmic, nucleo-cytoplasmic and plasma membrane proteins all showed similar distributions while the nuclear proteins appeared slightly less likely to be restricted to 10 or fewer tissues (p < = 0.28). Despite the observed differences in distributions, only the difference between kinase types appears to be statistically significant using Chi squared tests for equality of distributions (p < = 0.02).

### Integrated interface

PhosphoregDB is based on a customised version of the mouse protein sub-cellular localization database LOCATE [34,35]. In addition to expression and localization information, we have also provided extensive cross references into other relevant data sources. Kinase specific resources include links into the PhosphoELM database of reported kinase substrate relationships [36,37] and the Protein Kinase Resource (PKR) integrated database of protein kinase sequence, structure and domains [38]. We also place the enzymes in the context of pathways by mappings into the Kyoto Encyclopedia of Genes and Genomes [39,40]. Further potential substrate and interaction relationships are extracted from the public protein:protein interaction databases BIND and MINT [41-44]. For each of these associations, the nature of the interaction is recorded, and a link provided to the original database.

**Figure 6:**
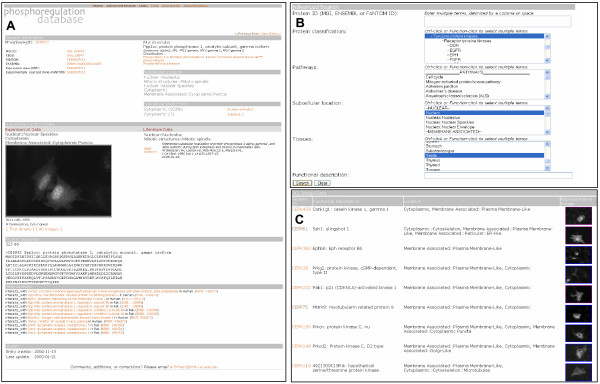
**PhosphoregDB interface**. **A**) Example entry for Ppp1cc **B**) Advanced Query interface demonstrating a search for Tyrosine kinases, localizing to the nucleus and expressed in testis **C**) Results of a batch search for "membrane associated" phosphoregulators with experimental support.

Throughout the database MGI nomenclature is used to ensure consistent use of gene symbols [45]. Users can either use a simple query by symbol, synonym or keyword to retrieve entries or they can use the advanced search options. On the advanced page users can query by protein identifier (MGI, ENSEMBL, FANTOMDB [18,45,46]), classification, pathway, sub-cellular location or tissue. Alternatively more complex queries combining all of the above can be formed (for example: return all tyrosine kinases that are localized to the nucleus and expressed in testis) (Fig. 6).

## Conclusion

By combining classification, expression and sub-cellular localization of all protein kinases and phosphatases we have produced a useful resource for querying the global characteristics of these components. It allows users to query where these molecules are expressed at both the sub-cellular and tissue resolution and reveals higher order relationships between expression, classification and localization. The observation of higher tissue specificity for tyrosine kinases has been suggested previously by the observed lack of these proteins in yeast and their expansion in metazoans [9,47]. However a system wide review of their expression and localization has not previously been described. Despite the observation that tyrosine specific kinases are more likely to be restricted in expression, the trend is more subtle than that and we observe both ubiquitous and restricted expression patterns for kinases and phosphatases from all classes and sub-cellular localizations.

The observed tissue restriction for these proteins has important implications for modelling the system and for elucidating substrate-enzyme relationships. Only 30% of the enzymes were detected in all 61 tissues of the GNF gene atlas, leaving 70% with some level of tissue restriction. When attempting to identify the kinase or phosphatase responsible for a given activity, the tissue restriction of the target and candidate enzyme should be considered. With the move towards systems biology, models need to consider whether they are dependent upon tissue specific components and in so doing report the cell types for which the model is valid.

Finally, we have highlighted the need to consider physical co-location of enzyme and substrate at the level of tissue distribution and sub-cellular compartment in designing realistic models of these systems. However dynamic changes in sub-cellular distributions add another aspect of complexity to the system. Regulation of sub-cellular localization, by post-translational modifications including phosphorylation is used to regulate access of enzyme to pools of substrate [48,49]. The next stage in this process will be to assess these dynamic changes in localization upon various stimuli and during the cell cycle.

## Methods

### Population of PhosphoregDB

There have been two previous attempts at defining the kinase-like gene complement of mouse [7,8]. In 2003 we used InterProScan [50] to identify 561 peptides containing predicted kinase catalytic domain motifs. In the previous study we included ENSEMBL predictions as well as those with transcript evidence. In 2004, Caenepeel et al. used a combination of BLAST, gene predictions and hidden markov models to identify 540 kinase-like sequences and 97 kinase-like pseudogenes. We have reviewed both datasets and mapped them to the mouse genome (mm5: may 2004 assembly). False positives from duplicate entries, withdrawn ENSEMBL predictions and the Prosite motif PS000107 were removed. Additionally the A6/twinfilins were not included as their reported novel tyrosine kinase activity has not been reproduced [51,52].

The 526-kinase like sequences we consider within this paper correspond to transcripts that map uniquely to the genome and for which there is EST evidence. Similarly for the protein phosphatase-like loci we analyse the set of 158 for which there is transcript evidence. A table summarizing the loci studied is provided as supplementary material 1. This includes unique identifier, description, genomic co-ordinates, MGI symbol, representative nucleotide accession, GNF gene atlas probeID and a comparison to the previous datasets.

### Generation of fragments for overlap fusion PCR

Linear expression constructs were generated by fusing three PCR generated fragments; a CMV promoter, a *myc *9E11 epitope tagged open reading frame and a dual SV40 terminator fragment (Fig. 1A). Full length kinase and phosphatase open reading frames identified in the FANTOM2 project [17] were amplified using a vector overlap primer (P8/P15) and a gene overlap primer GSN that introduced a 5aa sequence from VP16 in frame with the CDS to be tested [53].

N-terminal promoter fragments were amplified from pRNprom using the primer pair AL41 and AL34. Similarly the SV40 terminator fragment was amplified from pRNterm using AL25 and AL37. For the C-terminal system, primer pairs (AL19&AL34) and (AL40&AL37) were used to amplify the promoter and myc-terminator fragments from pCCprom and pCCterm respectively. These fragments were amplified with Triplemaster polymerase (Eppendorf) and agarose gel purified (QIAquick gel extraction kit), quantified and stored in aliquots at 10 fmol/μl. The N-terminal system was used to determine the sub-cellular localization of 157 open reading frames while three open reading frames with n-terminal features [DDBJ:AK018144, DDBJ:AK029057, DDBJ:AK031968] a c-terminal system tagging system was used. (Supplementary figure).

### Overlap Fusion PCR

Linear expression constructs were generated by combining 10 fmol of each of the terminator and promoter fragments with 1 μl of the gene specific PCR (50–500 ng) in a 25 μl high fidelity PCR reaction using the Triplemaster PCR system (Eppendorf) and the following cycle conditions (95-30", 52-30", 72-2'00"). For a fusion PCR product to be generated the promoter and terminator fragments must prime off the gene specific PCR fragment. The nested primers AL35 and AL38, which are directed towards the 5' of the promoter fragment and 3' of the terminator fragment respectively, are then used to amplify the full-length product further (Fig. 1). PCR products were checked by agarose gel electrophoresis (0.8% agarose, 1× TAE). Products that converted shift to a size equal to the sum of the sizes of the promoter, gene of interest and terminator fragments.

### Generation of promoter and terminator clones pRNprom, pRNterm, pCCprom, & pCCterm

Promoter and terminator sequences were amplified from pEGFP-C1 and pEGFP-N1 (Clontech) using long primers that incorporated the framework for a nested primer design and the overlap sequences necessary for fusion PCR (supplementary data 3). The N-terminal system promoter-myc fragment was amplified from pEGFP-C1 using primers AL33 and AL41, while the terminator fragment was amplified from pEGFP-C1 using primers AL36 and AL25. The C-terminal system promoter and myc-terminator fragments were amplified from pEGFP-N1 using primer pairs AL33 and AL19, and AL36 and AL40 respectively (Supplementary fig. 1). These fragments were subcloned into pGEMT (Promega) and sequence verified. The constructs for the N-term and C-term systems are designated pRNprom, pRNterm, pCCprom, pCCterm respectively, where the prom and term refer to the promoter and terminator fragments.

### Cell-culture and transfections

HeLa cells were grown in DMEM (Invitrogen) supplemented with 5% FCS (Trace). The day before transfection, 24 well plates containing 10 mm coverslips were seeded with approximately 20000 cells/well. PCR generated linear expression constructs were transfected using Effectene (QIAGEN). 1 μl of crude PCR product (approximately 50 ng-500 ng) was added to 3 μl of EC buffer/enhancer mix (1:10 Enhancer:EC buffer). Gently vortexed, incubated at room temperature for 5 minutes, 1 μl of effectene was added and again gently vortexed and incubated at room temperature for at least 10 minutes. These transfection mixes were then mixed with 50 μl media and added to coverslips. Transfections were left for 16–18 hrs prior to harvest.

### Immunofluorescence

Coverslips were washed 3 times with PBS, fixed with 4% formaldehyde for 20 minutes, washed 3 times in PBS, permeabilized with 0.05% Triton X-100 for 15 minutes, washed again with PBS and then blocked in 2% BSA for 1 hr. Coverslips were then incubated for 1 hr at room temperature with anti-*myc *9E11 (1:4000 dilution in 2% BSA; Cell Signalling Technologies). Coverslips were then washed 3 times with 2% BSA in PBS and then incubated for 30 minutes in the dark with Cy3 labelled anti-mouse secondary (1:500 dilution in 2% BSA; Zymed). Finally coverslips were counterstained with DAPI (ICN) washed 3 times in 2% BSA, 3 times in PBS and mounted with Movial (Calbiochem). Note: we also generated and tested N and C-terminal GFP systems but found the nucleo-cytoplasmic distribution of native GFP problematic for accurately determining localizations. For this reason we chose the *myc *9E11 epitope tag which lacks any distinguishable targeting capacity and negligible changes to fusion protein size.

### Microscopy

Multiple fluorescence images were acquired for each construct using NIH image on an Olympus AX70 camera at 100× magnification with oil immersion. After acquisition TIF images were converted to JPG format for web-based publishing (original TIF images are archived). No further manipulations, or threshold was applied to grey scale fluorescent images.

### Consensus localization of all protein kinases and phosphatases

Combining, experimentally observed localizations, publicly recorded localizations and predictions by Proteome Analyst and SubLocV1.0 [19,22,25,26] we produced consensus localizations for every protein kinase and phosphatase. Experimentally derived localizations were accepted in preference to bioinformatic predictions. For all our experimental localizations the open reading frame displayed is the actual ORF tested (ie. a direct relationship between localization and image). For this reason in the few cases where there was a discrepancy between the published localization and our localization, ours was chosen as we could not ensure the published localization referred to exactly the same peptide sequence. However in cases where the publications gave greater detail (eg. Endosomal as opposed to cytoplasmic puncta) we accepted the published localization over ours. For the remaining untested sequences, Proteome Analyst predictions were accepted over SubLoc predictions. Additionally membrane organization was predicted using signalP3.0 and TMHMM [20,21,23,24] and nuclear localization signals and nuclear export sequences were identified using NetNES, the prosite bipartite nuclear localization motif PS00015 and predictNLS [28-33]. Additionally we provide links into a public database of sub-cellular localizations DBSubLoc [16,54]. For every entry, all available evidence used to make a decision on localization is presented to the user.

### Tissue specific expression of the kinome and phosphatome

In a similar strategy to that described recently for correlating the expression profiles of components of Rab GTPase Trafficking Networks [55] we extracted GC-RMA normalised expression data from a panel of 61 normal mouse tissues for 649 kinase and phosphatase loci from the Genomics Institute of the Novartis Research Foundation gene atlas [14,15]. To assess tissue specificity we used two approaches, the first involved hierarchical clustering of the dataset and the second involved absolute counts of how many tissues a given gene was detected in. For both a threshold of 200 relative expression units was used to define a probe as having been detected in a given tissue [15]. Using this threshold 571 loci had detectable signal in at least one tissue.

Hierarchical clustering was carried out using Genespring 6.2. Briefly for each loci with multiple probes a representative probe was chosen by taking the probe detected in the highest number of tissues (and if the same then the highest median signal). This GC-RMA normalised data was then median centred in Genespring and hierarchical gene and tissue trees were generated using Pearson-correlation.

## Authors' contributions

Experiments were conceived by AF, SG, HS and YH. Materials used in the experimental localizations were generated by AF, MG, CF, HS, MK and CK. Database was designed and implemented by LF, DT, AF and RT. Manuscript was written by AF and SG.

## Supplementary Material

Additional File 1 **Definition of dataset**. Excel file containing descriptions and mappings of all kinase-like and phosphatase-like sequences in PhosphoregDB. MGI_ID, kinome_id, Description, representative GNF_probe Click here for file

Additional File 2 **Classification tree for phosphoregDB**. Excel file containing the classifications used in phosphoregDB Click here for file

Additional File 3 **Localization data incorporated into phosphoregDB**. Excel file of the localizations Click here for file

Additional File 4 **NES and NLS predictions, and pubmed entries reporting nucleocytoplasmic shuttling**. Excel file of kinases and phosphatases with predicted nuclear localization signals, nuclear export sequences or reported to undergo nucleocytoplasmic shuttling. Click here for file

Additional File 5 **Primers used for overlap PCRs and c-terminal tagging schematic**. PDF file showing the c-terminal tagging system Click here for file

Additional File 6 **GNF Probes used to measure expression in phosphoregDB**. Excel file showing the mapping of phosphoregulator IDs to GNF probes and GC-RMA normalised data. Representative probes are flagged and number of samples with signal above 200 provided Click here for file

Additional File 7 **All supplementary data**. Zip file containing all of the additional data files 1, 2, 3, 4, 5, 6 Click here for file

## References

[B1] Hunter T (1998). The Croonian Lecture 1997. The phosphorylation of proteins on tyrosine: its role in cell growth and disease. Philos Trans R Soc Lond B Biol Sci.

[B2] Bubulya PA, Prasanth KV, Deerinck TJ, Gerlich D, Beaudouin J, Ellisman MH, Ellenberg J, Spector DL (2004). Hypophosphorylated SR splicing factors transiently localize around active nucleolar organizing regions in telophase daughter nuclei. J Cell Biol.

[B3] Ding Y, Dale T (2002). Wnt signal transduction: kinase cogs in a nano-machine?. Trends Biochem Sci.

[B4] Yaffe MB (2002). Phosphotyrosine-binding domains in signal transduction. Nat Rev Mol Cell Biol.

[B5] Penrose KJ, Garcia-Alai M, de Prat-Gay G, McBride AA (2004). Casein Kinase II phosphorylation-induced conformational switch triggers degradation of the papillomavirus E2 protein. J Biol Chem.

[B6] McCoy CE, Campbell DG, Deak M, Bloomberg GB, Arthur JS (2005). MSK1 activity is controlled by multiple phosphorylation sites. Biochem J.

[B7] Caenepeel S, Charydczak G, Sudarsanam S, Hunter T, Manning G (2004). The mouse kinome: discovery and comparative genomics of all mouse protein kinases. Proc Natl Acad Sci U S A.

[B8] Forrest AR, Ravasi T, Taylor D, Huber T, Hume DA, Grimmond S (2003). Phosphoregulators: protein kinases and protein phosphatases of mouse. Genome Res.

[B9] Hunter T, Plowman GD (1997). The protein kinases of budding yeast: six score and more. Trends Biochem Sci.

[B10] Yang DD, Conze D, Whitmarsh AJ, Barrett T, Davis RJ, Rincon M, Flavell RA (1998). Differentiation of CD4+ T cells to Th1 cells requires MAP kinase JNK2. Immunity.

[B11] Sweatt JD (2001). The neuronal MAP kinase cascade: a biochemical signal integration system subserving synaptic plasticity and memory. J Neurochem.

[B12] Cowley S, Paterson H, Kemp P, Marshall CJ (1994). Activation of MAP kinase kinase is necessary and sufficient for PC12 differentiation and for transformation of NIH 3T3 cells. Cell.

[B13] Johnson SA, Hunter T (2005). Kinomics: methods for deciphering the kinome. Nat Methods.

[B14] GNF gene expression atlas. http://symatlas.gnf.org.

[B15] Su AI, Wiltshire T, Batalov S, Lapp H, Ching KA, Block D, Zhang J, Soden R, Hayakawa M, Kreiman G, Cooke MP, Walker JR, Hogenesch JB (2004). A gene atlas of the mouse and human protein-encoding transcriptomes. Proc Natl Acad Sci U S A.

[B16] Guo T, Hua S, Ji X, Sun Z (2004). DBSubLoc: database of protein subcellular localization. Nucleic Acids Res.

[B17] Okazaki Y, Furuno M, Kasukawa T, Adachi J, Bono H, Kondo S, Nikaido I, Osato N, Saito R, Suzuki H, Yamanaka I, Kiyosawa H, Yagi K, Tomaru Y, Hasegawa Y, Nogami A, Schonbach C, Gojobori T, Baldarelli R, Hill DP, Bult C, Hume DA, Quackenbush J, Schriml LM, Kanapin A, Matsuda H, Batalov S, Beisel KW, Blake JA, Bradt D, Brusic V, Chothia C, Corbani LE, Cousins S, Dalla E, Dragani TA, Fletcher CF, Forrest A, Frazer KS, Gaasterland T, Gariboldi M, Gissi C, Godzik A, Gough J, Grimmond S, Gustincich S, Hirokawa N, Jackson IJ, Jarvis ED, Kanai A, Kawaji H, Kawasawa Y, Kedzierski RM, King BL, Konagaya A, Kurochkin IV, Lee Y, Lenhard B, Lyons PA, Maglott DR, Maltais L, Marchionni L, McKenzie L, Miki H, Nagashima T, Numata K, Okido T, Pavan WJ, Pertea G, Pesole G, Petrovsky N, Pillai R, Pontius JU, Qi D, Ramachandran S, Ravasi T, Reed JC, Reed DJ, Reid J, Ring BZ, Ringwald M, Sandelin A, Schneider C, Semple CA, Setou M, Shimada K, Sultana R, Takenaka Y, Taylor MS, Teasdale RD, Tomita M, Verardo R, Wagner L, Wahlestedt C, Wang Y, Watanabe Y, Wells C, Wilming LG, Wynshaw-Boris A, Yanagisawa M, Yang I, Yang L, Yuan Z, Zavolan M, Zhu Y, Zimmer A, Carninci P, Hayatsu N, Hirozane-Kishikawa T, Konno H, Nakamura M, Sakazume N, Sato K, Shiraki T, Waki K, Kawai J, Aizawa K, Arakawa T, Fukuda S, Hara A, Hashizume W, Imotani K, Ishii Y, Itoh M, Kagawa I, Miyazaki A, Sakai K, Sasaki D, Shibata K, Shinagawa A, Yasunishi A, Yoshino M, Waterston R, Lander ES, Rogers J, Birney E, Hayashizaki Y (2002). Analysis of the mouse transcriptome based on functional annotation of 60,770 full-length cDNAs. Nature.

[B18] FANTOM2 DB. http://fantom2.gsc.riken.go.jp/db/.

[B19] Szafron D, Lu P, Greiner R, Wishart DS, Poulin B, Eisner R, Lu Z, Anvik J, Macdonell C, Fyshe A, Meeuwis D (2004). Proteome Analyst: custom predictions with explanations in a web-based tool for high-throughput proteome annotations. Nucleic Acids Res.

[B20] Bendtsen JD, Nielsen H, von Heijne G, Brunak S (2004). Improved prediction of signal peptides: SignalP 3.0. J Mol Biol.

[B21] Krogh A, Larsson B, von Heijne G, Sonnhammer EL (2001). Predicting transmembrane protein topology with a hidden Markov model: application to complete genomes. J Mol Biol.

[B22] Hua S, Sun Z (2001). Support vector machine approach for protein subcellular localization prediction. Bioinformatics.

[B23] TMHMM transmembrane region predictor. http://www.cbs.dtu.dk/services/TMHMM/.

[B24] SignalP signal peptide predictor. http://www.cbs.dtu.dk/services/SignalP/.

[B25] PENCE Proteome Analyst. http://www.cs.ualberta.ca/~bioinfo/PA/.

[B26] SubLocv1.0. http://www.bioinfo.tsinghua.edu.cn/SubLoc/eu_predict.htm.

[B27] Fornerod M, Ohno M, Yoshida M, Mattaj IW (1997). CRM1 is an export receptor for leucine-rich nuclear export signals. Cell.

[B28] Gattiker A, Gasteiger E, Bairoch A (2002). ScanProsite: a reference implementation of a PROSITE scanning tool. Appl Bioinformatics.

[B29] Cokol M, Nair R, Rost B (2000). Finding nuclear localization signals. EMBO Rep.

[B30] la Cour T, Kiemer L, Molgaard A, Gupta R, Skriver K, Brunak S (2004). Analysis and prediction of leucine-rich nuclear export signals. Protein Eng Des Sel.

[B31] NetNES nuclear export sequence predictor. http://www.cbs.dtu.dk/services/NetNES/.

[B32] PredictNLS: Prediction of NLSs. http://cubic.bioc.columbia.edu/predictNLS/.

[B33] Prosite bipartite NLS. http://www.expasy.org/cgi-bin/nicesite.pl?PS00015.

[B34] Fink JL, Aturaliya RN, Davis MJ, Zhang F, Hanson K, Teasdale MS, Kai C, Kawai J, Carninci P, Hayashizaki Y, Teasdale RD (2006). LOCATE: a mouse protein subcellular localization database. Nucleic Acids Res.

[B35] LOCATE: Mouse Protein localization database. http://locate.imb.uq.edu.au.

[B36] Diella F, Cameron S, Gemund C, Linding R, Via A, Kuster B, Sicheritz-Ponten T, Blom N, Gibson TJ (2004). Phospho.ELM: a database of experimentally verified phosphorylation sites in eukaryotic proteins. BMC Bioinformatics.

[B37] Phospho.ELM: The Protein Phosphorylation Database. http://phospho.elm.eu.org/.

[B38] PKR: The Protein Kinase Resource. http://www.kinasenet.org/pkr/Welcome.do.

[B39] Kanehisa M, Goto S, Kawashima S, Okuno Y, Hattori M (2004). The KEGG resource for deciphering the genome. Nucleic Acids Res.

[B40] KEGG: Kyoto Encyclopedia of Genes and Genomes. http://www.genome.ad.jp/kegg/.

[B41] BIND: the Biomolecular Interaction Network Database. http://bind.ca/.

[B42] MINT a Molecular INTeraction database.

[B43] Bader GD, Betel D, Hogue CW (2003). BIND: the Biomolecular Interaction Network Database. Nucleic Acids Res.

[B44] Zanzoni A, Montecchi-Palazzi L, Quondam M, Ausiello G, Helmer-Citterich M, Cesareni G (2002). MINT: a Molecular INTeraction database. FEBS Lett.

[B45] Mouse Genome Database (MGD). http://www.informatics.jax.org/.

[B46] Ensembl Mouse Genome Server. http://www.ensembl.org/Mus_musculus/.

[B47] Manning G, Plowman GD, Hunter T, Sudarsanam S (2002). Evolution of protein kinase signaling from yeast to man. Trends Biochem Sci.

[B48] Brennan JA, Volle DJ, Chaika OV, Lewis RE (2002). Phosphorylation regulates the nucleocytoplasmic distribution of kinase suppressor of Ras. J Biol Chem.

[B49] Katoh Y, Takemori H, Min L, Muraoka M, Doi J, Horike N, Okamoto M (2004). Salt-inducible kinase-1 represses cAMP response element-binding protein activity both in the nucleus and in the cytoplasm. Eur J Biochem.

[B50] Quevillon E, Silventoinen V, Pillai S, Harte N, Mulder N, Apweiler R, Lopez R (2005). InterProScan: protein domains identifier. Nucleic Acids Res.

[B51] Beeler JF, LaRochelle WJ, Chedid M, Tronick SR, Aaronson SA (1994). Prokaryotic expression cloning of a novel human tyrosine kinase. Mol Cell Biol.

[B52] Vartiainen M, Ojala PJ, Auvinen P, Peranen J, Lappalainen P (2000). Mouse A6/twinfilin is an actin monomer-binding protein that localizes to the regions of rapid actin dynamics. Mol Cell Biol.

[B53] Suzuki H, Fukunishi Y, Kagawa I, Saito R, Oda H, Endo T, Kondo S, Bono H, Okazaki Y, Hayashizaki Y (2001). Protein-protein interaction panel using mouse full-length cDNAs. Genome Res.

[B54] DBSubLoc Localization Database. http://www.bioinfo.tsinghua.edu.cn/dbsubloc.html.

[B55] Gurkan C, Lapp H, Alory C, Su AI, Hogenesch JB, Balch WE (2005). Large-scale profiling of Rab GTPase trafficking networks: the membrome. Mol Biol Cell.

