# Supplementary material for: PhosphoregDB: The tissue and sub-cellular distribution of mammalian protein kinases and phosphatases
Source: BMC Bioinformatics. 2006 Feb 20;7:82. doi: 10.1186/1471-2105-7-82 (PMC1395337; doi:10.1186/1471-2105-7-82)
Supplement: Additional File 5 — Primers used for overlap PCRs and c-terminal tagging schematic. PDF file showing the c-terminal tagging system [file 1471-2105-7-82-S5.pdf]

**Supplementary table1**: Primer sequences used in the fusion PCR technique

| Oligo name | Primers for Gene specific products | Description |
|---|---|---|
| GSN1 | GAAGGAGCCGCCACCATG(X)n | In frame gene-specific overlap primer for N-terminal tagging |
| GSC1 | ATCCTCTTCTGATATCAGCTTCTGTTC(X)n | In frame gene-specific overlap primer for C-terminal tagging |
| P8 | AGCGGATAACAATTTCACACAGGAAAC | Vector specific primer used for FANTOM clones in N-term system |
| P15 | AGCGGATAACAATTTCACACAGGAAACCGCCAGGGTTTTCCCAGTCACGAC | Vector specific primer used for ZA series FANTOM clones in N-term system |
| AL18 | GGACTCAGATCTCGAGCTCAAGGAATTGTAATACGACTCACTATAGG | Vector specific primer used for FANTOM clones in N-term system |
| AL28 | GGACTCAGATCTCGAGCTCAAGGAAATTAACCCTCACTAAAGGG | Vector specific primer used for ZA series FANTOM clones in N-term system |
| | | |
| Primers for promoter fragments | | |
| AL33 | GACGCGCTTCTGCTTCGAGACTCGCTTACCTGATGCTGCGTTATCCCCTGATTCTGTGGA | Used to introduce primer sequences AL34 and AL35 to promoter casette from EGFP-C1 |
| AL34 | GACGCGCTTCTGCTTCGAGAC | Used to amplify the promoter fragment |
| AL35 | GACTCGCTTACCTGATGCTGC | Nested primer used to prime off the promoter fragment during the fusion PCR step |
| AL1 | AACCGTATTACCGCCATGCAT | Additional promoter fragment nested primer for reamplification of weak products |
| AL41 | CATGGTGGCGGCTCCTTCCAGATCCTCTTCTGATATCAGCTTCTGTTCCATGGTGGCGACCGGTAGCGCTAG | Used to introduce an n-terminal myc tag onto the CMV promoter cassette from EGFP-C1 |
| AL19 | CTTGAGCTCGAGATCTGAGTCC | Used to amplify the CMV promoter cassette from EGFP-C1 |
| | | |
| Primers for terminator fragments | | |
| AL36 | GGCTCTTGCATGGTTTGACGACGTGCTCAGTACGCTGGACAAACCACAACTAGAATGC | Used to introduce primer sequences AL37 and AL38 to terminator casette from EGFP-C1 |
| AL37 | GGCTCTTGCATGGTTTGACG | Used to amplify the terminator fragment |
| AL38 | CGACGTGCTCAGTACGCTGGA | Nested primer used to prime off the terminator fragment during the fusion PCR step |
| AL5 | GGACAAACCACAACTAGAATGC | Additional terminator fragment nested primer for reamplification of weak products |
| AL25 | GTTTCCTGTGTGAAATTGTTATCCGCTCGAATTCTGCAGTCGACGGTACC | nterm vec overlap for term |
| AL40 | GAACAGAAGCTGATATCAGAAGAGGATCTGtagCGAATTCTGCAGTCGACGGTACC | cterm sys myc term |

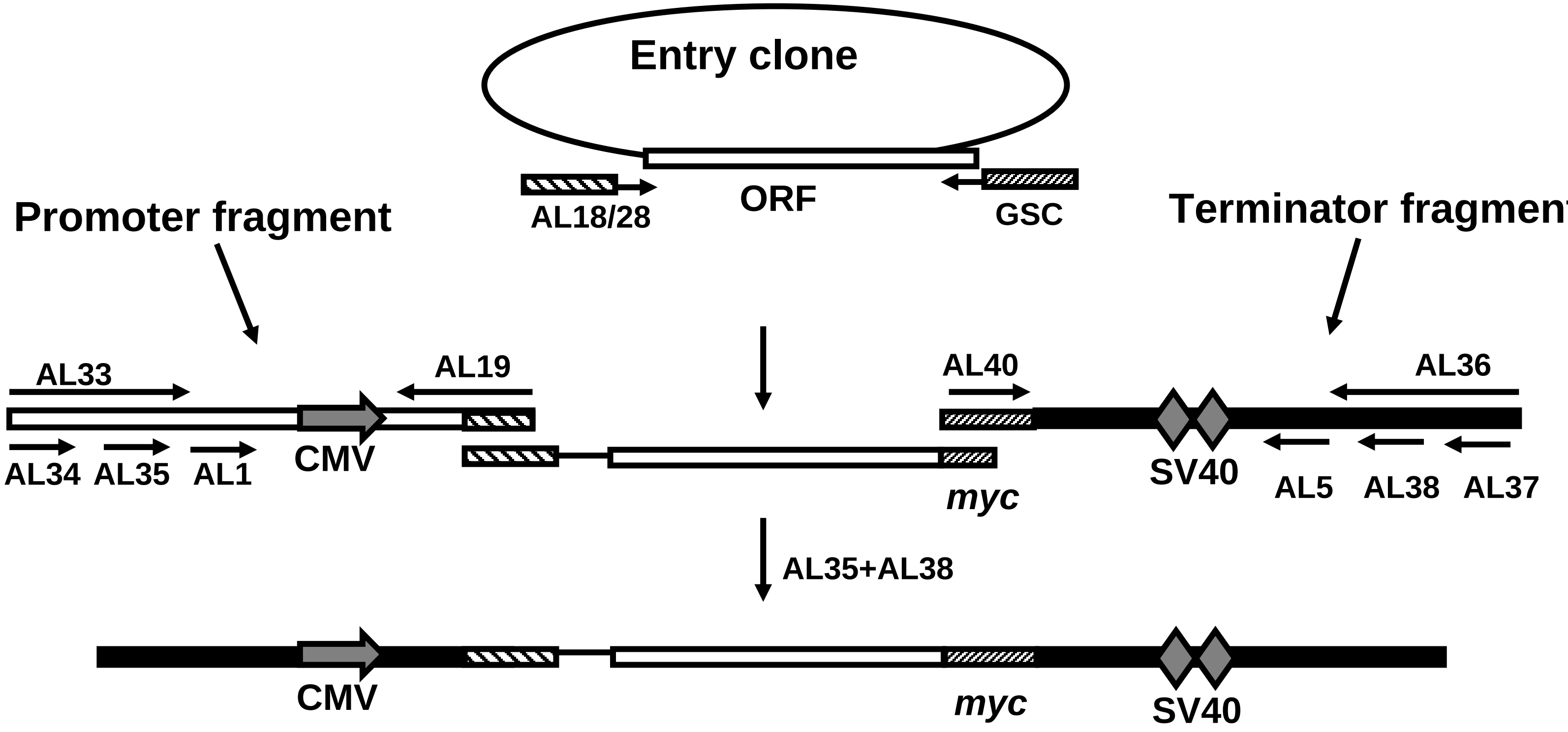


Supplementary figure 1: Generation of c-terminally tagged open reading frames
